# STAT1 Signaling in Astrocytes Is Essential for Control of Infection in the Central Nervous System

**DOI:** 10.1128/mBio.01881-16

**Published:** 2016-11-08

**Authors:** Shinya Hidano, Louise M. Randall, Lucas Dawson, Hans K. Dietrich, Christoph Konradt, Peter J. Klover, Beena John, Tajie H. Harris, Qun Fang, Bradley Turek, Takashi Kobayashi, Lothar Hennighausen, Daniel P. Beiting, Anita A. Koshy, Christopher A. Hunter

**Affiliations:** a Department of Pathobiology, School of Veterinary Medicine, University of Pennsylvania, Philadelphia, Pennsylvania, USA; b Department of Medicine, University of Melbourne, Peter Doherty Institute, Parkville, Victoria, Australia; c Department of Neurology, Department of Immunobiology, BIO5 Institute, University of Arizona, Tucson, Arizona, USA; d Laboratory of Genetics and Physiology, National Institute of Diabetes, Digestive and Kidney Diseases, National Institutes of Health, Bethesda, Maryland, USA; e Center for Brain Immunology and Glia (BIG), Department of Neuroscience, University of Virginia, Charlottesville, Virginia, USA; f Department of Infectious Disease Control, Faculty of Medicine, Oita University, Yufu, Oita, Japan

## Abstract

The local production of gamma interferon (IFN-γ) is important to control *Toxoplasma gondii* in the brain, but the basis for these protective effects is not fully understood. The studies presented here reveal that the ability of IFN-γ to inhibit parasite replication in astrocytes *in vitro* is dependent on signal transducer and activator of transcription 1 (STAT1) and that mice that specifically lack STAT1 in astrocytes are unable to limit parasite replication in the central nervous system (CNS). This susceptibility is associated with a loss of antimicrobial pathways and increased cyst formation in astrocytes. These results identify a critical role for astrocytes in limiting the replication of an important opportunistic pathogen.

## IMPORTANCE

Astrocytes are the most numerous cell type in the brain, and they are activated in response to many types of neuroinflammation, but their function in the control of CNS-specific infection is unclear. The parasite *Toxoplasma gondii* is one of the few clinically relevant microorganisms that naturally infects astrocytes, and the studies presented here establish that the ability of astrocytes to inhibit parasite replication is essential for the local control of this opportunistic pathogen. Together, these studies establish a key role for astrocytes as effector cells and in the coordination of many aspects of the protective immune response that operates in the brain.

## INTRODUCTION

Astrocytes are considered the predominant subtype of glial cells in the brain, which provide support and protection for neurons (1–4). They also have a role in the maintenance of the blood-brain barrier, and reactive astrocytes are a feature of the local response to traumas that affect the central nervous system (CNS) (5). While astrocytes can be infected by many neurotropic viruses, there are a limited number of bacterial or eukaryotic pathogens that can replicate in these cells, and *Toxoplasma gondii* is perhaps the most common clinically relevant organism that infects these glia. In murine models of toxoplasmic encephalitis (TE), astrocyte activation and proliferation are prominent, and these cells produce chemokines that can influence the recruitment of T cells and dendritic cells (DCs) as well as microglial cell activation (5, 6). Furthermore, consistent with the role of gamma interferon (IFN-γ) as the major mediator of resistance to TE (7, 8), *in vitro* stimulation of astrocytes with IFN-γ leads to an inhibition of parasite replication that is dependent on the p47 GTPases IFN-γ-induced GTPase (IGTP) and interferon-inducible GTPase 1 (IIGP1) and p65 GTPase guanine nucleotide binding protein 2 (GBP2) (9–13). In addition, IFN-γ has been shown to promote astrocyte production of multiple cytokines and chemokines, in particular CXCL10, which are involved in the recruitment of T cells and macrophages into the brain for the local control of *T. gondii* (6, 14–17).

The ability to directly address the role of astrocytes in the pathogenesis of TE has been a challenge (18). However, in mice that lack glial fibrillary acid protein (GFAP), an intermediate filament expressed by astrocytes and upregulated after activation, challenge with *Staphylococcus aureus* or *T. gondii* is associated with a “reduced bordering function” and an inability to restrict inflammation, which leads to pathogen spread (19). Studies using mice with lineage-specific deletion of gp130 in astrocytes showed that this cytokine receptor was not intrinsically required for the ability of these cells to control *T. gondii* but was required to restrict inflammation and prevent astrocyte apoptosis (20). Similarly, the loss of transforming growth factor β (TGF-β) signaling in astrocytes does not affect parasite burden but results in increased CNS inflammation (21). Together, these previous studies emphasize the critical role for astrocytes in limiting CNS inflammation during TE but do not address whether astrocytes have a role in direct parasite control or in promoting local antiparasite responses. Perhaps the most prominent pathway for control of *T. gondii* in the CNS is mediated by IFN-γ *in vivo* (8, 22), and this is a cytokine that can activate astrocytes *in vitro* to limit parasite replication (9–11). In an attempt to understand the role of astrocytes in the control of *T. gondii*, a series of *in vitro* and *in vivo* approaches were used to assess how loss of STAT1 affected astrocyte function. This pathway was chosen because IFN-γ signals through this transcription factor, which is essential for acute resistance to *T. gondii* (23, 24). Furthermore, the ability of *T. gondii* to interfere with STAT1 activity suggests that this pathway has an important role in parasite survival (23, 25–31). The studies presented here highlight the critical importance of astrocytes in the direct control of *T. gondii* in the CNS and provide new insights into their role in coordinating antiparasite effector activities.

## RESULTS

### Activation of STAT1 in astrocytes promotes the control of *T. gondii.*

**FIG 1:**
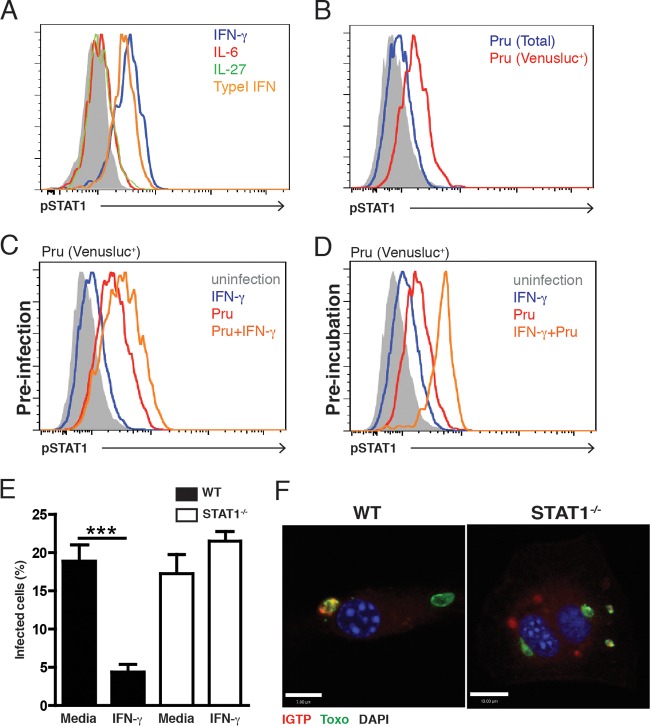
Astrocyte expression of STAT1 is required for IFN-γ-mediated control of parasite replication. (A) Primary astrocytes were stimulated with IFN-γ (100 U/ml), type I IFN (100 U/ml), IL-6 (10 ng/ml), or IL-27 (10 ng/ml) for 1 h and then assayed for phospho-STAT1. (B) Cells were infected with the Pru-Venusluc strain of *T. gondii* (parasite/cell ratio, 1:1) for 24 h and then assayed for phospho-STAT1. (C) Cells were infected with Pru-Venusluc for 24 h, stimulated with IFN-γ for 20 h, and then assayed for phospho-STAT1. (D) Cells were stimulated with 100 U/ml IFN-γ before the Pru-Venusluc strain of *T. gondii* (parasite/cell ratio, 1:1) for 24 h and then assayed for STAT1 phosphorylation by FACS. (E) To assay the ability of astrocytes to limit parasite growth, cells were stimulated with IFN-γ (100 U/ml) for 24 h and then infected with *T. gondii* (parasite/cell ratio, 1:1), and the percentage of infected cells was assessed microscopically after 20 h. (F) Cells from WT or STAT1^−/−^ mice were stimulated with 100 U/ml IFN-γ before the Pru-GFP strain of *T. gondii* for 1.5 h and stained for *T. gondii* Pru-GFP (green) and anti-IGTP (red) antibodies, with DAPI (blue) as a nuclear counterstain. Bars, 7 µm (WT) and 13 µm (STAT1^−/−^).

Although STAT1 signaling is activated in response to *T. gondii* infection in macrophages and other cell types, there are multiple reports that *T. gondii* modulates this pathway (25–27, 29, 30, 32), but whether this is relevant to astrocytes is unclear. Therefore, *in vitro* experiments were performed to assess the stimuli that activated STAT1 in primary murine astrocytes and to determine whether this transcription factor was required for their antimicrobial activities. As expected, incubation of astrocytes with IFN-γ or type I IFNs for 2 h resulted in STAT1 phosphorylation (Fig. 1A) that was downregulated by 24 h (data not shown), whereas interleukin-6 (IL-6) or IL-27 (cytokines that signal through gp130 and which can engage JAK/STAT signaling) did not activate STAT1 (Fig. 1A). Infection of astrocytes with a Pru strain of *T. gondii* that expressed Venus (a modified version of yellow fluorescent protein [YFP]) failed to induce STAT1 phosphorylation at 2 h (data not shown), but by 24 h, there was a modest induction of STAT1 phosphorylation in the total population of cells in these cultures. By gating on the infected cells in these cultures (Pru-Venusluc^+^), it was apparent that this subpopulation had significant STAT1 phosphorylation (Fig. 1B). In similar experiments, analysis of cultures in which astrocytes were preinfected with *T. gondii* for 24 h and then stimulated with IFN-γ for 20 h revealed that infected cells contained phosphorylated STAT1 that was potentiated in the presence of IFN-γ Similarly, in cultures in which astrocytes were preincubated with IFN-γ prior to challenge with *T. gondii*, the infected cells (Pru-Venusluc^+^) showed the highest levels of STAT1 phosphorylation (Fig. 1C and D). Similar results were observed using the more virulent type I RH strain of *T. gondii* (data not shown). Thus, similarly to the effects in dendritic cells (31), infection of astrocytes with *T. gondii* results in the phosphorylation of STAT1 and the presence of IFN-γ (before or after infection) enhances these events. Next, the role of STAT1 in the ability of primary murine astrocytes to control *T. gondii* was assessed. Preincubation of wild-type (WT) astrocytes with IFN-γ (or type I IFN [data not shown]) for 24 h prior to infection resulted in a marked reduction in the percentage of infected cells present after 20 h, but this was not observed with STAT1^−/−^ astrocytes (Fig. 1E). The recruitment of the p47 GTPase IGTP to the parasitophorous vacuole of *T. gondii* is an important step in the ability of IFN-γ to activate astrocytes to limit parasite growth (9–11). Indeed, in astrocytes prestimulated with IFN-γ prior to infection, immunofluorescence assays revealed colocalization of IGTP with *T. gondii* (Fig. 1F). However, in the absence of STAT1, IGTP showed a diffuse pattern of staining and there was little colocalization with *T. gondii* (Fig. 1F). Together, these *in vitro* studies identify STAT1 as a key mediator of IFN-γ signaling required for the ability of astrocytes to control *T. gondii*.

### Generation and analysis of GFAP-Cre STAT1^f/f^ mice.

To assess the role of the STAT1 pathway in astrocytes in resistance to TE, mice expressing Cre recombinase under the control of a human GFAP promoter were crossed with STAT1^f/f^ mice to generate GFAP-Cre STAT1^f/f^ mice. While GFAP has been used as a specific marker of astrocytes, it can be expressed in other cell types that include microglial cells, neurons, and hepatic stellate cells in mice (33–35). Therefore, a series of studies were performed to determine the extent of STAT1 deletion in several of these populations. A comparison of primary astrocytes from control or GFAP-Cre STAT1^f/f^ neonates revealed that astrocytes from the GFAP-Cre STAT1^f/f^ mice had reduced STAT1 expression (see Fig. S1A and B in the supplemental material). In contrast, microglial cells isolated directly from the brains of adult control and GFAP-Cre STAT1^f/f^ mice and stimulated with IFN-γ had equivalent levels of STAT1 phosphorylation (see Fig. S1C). In addition, primary astrocytes from GFAP-Cre STAT1^f/f^ mice were unable to control the replication of *T. gondii* when stimulated with IFN-γ (see Fig. S1D), and astrocytes present in the brains of adult mice had reduced levels of STAT1 (see Fig. S1E).

**FIG 2:**
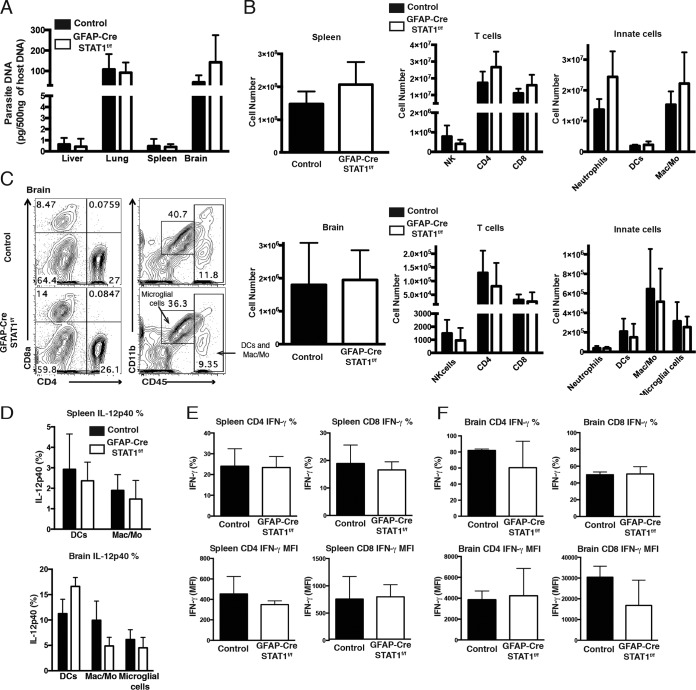
Comparison of the immune responses in acutely infected control and GFAP-Cre STAT1^f/f^ mice. (A) Real-time PCR specific for the *Toxoplasma* B1 repeat region was used to quantify the amount of parasite DNA from 500 ng of DNA purified from liver, lung, spleen, and brain at 10 dpi. (B) Mice were infected with *T. gondii*, and at 10 dpi, flow cytometry was used to estimate the absolute numbers of the indicated cell population in the spleen and brain from control (solid bars) and GFAP-Cre STAT1^f/f^ (open bars) mice. (C) Flow cytometric profiles of NK cells and CD4^+^ and CD8^+^ T cells and the proportions of neutrophils (CD45^hi^, Gr-1^+^, and CD11b^+^), DCs (CD45^hi^, Gr-1^−^, and CD11c^+^), macrophages/monocytes (CD45^hi^, Gr-1^−^, and CD11b^+^), and microglial cells (CD45^int^ and CD11b^+^) gated on dump (CD3, CD19, and NK1.1)^−^ in BMNCs from control and GFAP-Cre STAT1^f/f^ mice. Percentage of CD4^+^ or CD8^+^ T cells and neutrophils, DCs, macrophages/monocytes, and microglial cells of the gated dump-negative cells. (D) Frequency of IL-12p40 production by DCs, macrophages/monocytes, and microglial cells in the splenocytes and BMNCs after 4 h. (E and F) Frequency of IFN-γ production by CD4^+^ and CD8^+^ T cells in the spleen (E) and brain (F) after restimulation with PMA and ionomycin. Graphs show averages from a total of four mice per group at least three times with similar results.

Next, because GFAP is also expressed by hepatic stellate cells (36), studies were performed to compare the acute immune responses to *T. gondii* in control and GFAP-Cre STAT1^f/f^ mice. At day 10 postinfection, the parasite burdens in the liver, lung, spleen, and brain of these mice were similar (Fig. 2A; see also Fig. S2A in the supplemental material). A comparison of the immune cell populations in the periphery (spleen and lymph node) with the small numbers of brain mononuclear cells (BMNCs) that had started to accumulate in the CNS revealed that the control and GFAP-Cre STAT1^f/f^ mice had similar absolute numbers of neutrophils, DCs, macrophages/monocytes, natural killer (NK) cells, and T cells (Fig. 2B and C). DCs and macrophages are an early source of IL-12 required for resistance to *T. gondii* (37–42), and at day 10 postinfection, IL-12p40 production by myeloid cells in the spleen and that in the brain were comparable (Fig. 2D). Similarly, WT and GFAP-Cre STAT1^f/f^ mice developed comparable T cell responses, and there were no differences in the number of IFN-γ^+^ T cells or in the mean fluorescence intensity (MFI) for the levels of IFN-γ produced by these populations (Fig. 2E and F). Together, these studies indicate that the GFAP-Cre-driven deletion of STAT1 did not compromise generation of parasite-specific responses required or the initial STAT1-dependent control of *T. gondii*.

### GFAP-Cre STAT1^f/f^ mice are susceptible to toxoplasmic encephalitis.

**FIG 3:**
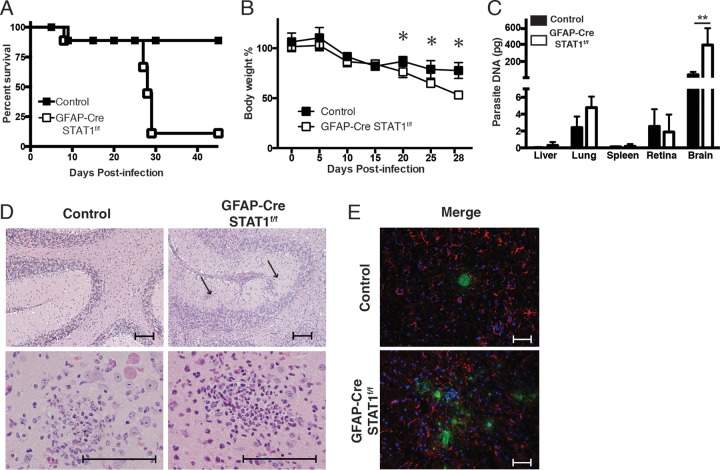
Increased susceptibility of GFAP-Cre STAT1^f/f^ mice to toxoplasmic encephalitis. (A and B) Control and GFAP-Cre STAT1^f/f^ mice were infected i.p. with 20 cysts of the ME49 strain of *T. gondii*, and survival (represented by Kaplan-Meier analysis) (A) and weight loss (B) were assessed. Data for five mice per group and a combination of two independent experiments are shown. (C) Real-time PCR specific for the *Toxoplasma* B1 repeat region was used to quantify the amount of parasite DNA from 500 ng of DNA purified from lung, liver, spleen, retina, and brain at 25 dpi. These graphs are means ± standard errors of the means and show the pooled averages from three independent experiments. *, *P* < 0.05; **, *P* < 0.01. (D) Representative hematoxylin-and-eosin-stained sections of the brains of control and GFAP-Cre STAT1^f/f^ mice infected for 25 days. Bar, 100 µm. (E) Frozen tissue sections from the brains of infected control and GFAP-Cre STAT1^f/f^ mice were used for IHC detection of *T. gondii* (green) or GFAP (red), with DAPI (blue) as a nuclear counterstain. Bar, 50 µm.

To assess the role of STAT1 in astrocyte function during TE, control and GFAP-Cre STAT1^f/f^ mice were infected with *T. gondii* and the course of disease was monitored. While the control mice were largely resistant, between 23 and 29 days postinfection (dpi) the GFAP-Cre STAT1^f/f^ mice succumbed to infection associated with marked weight loss (Fig. 3A and B). At 25 dpi, the parasite burdens (as assessed by parasite DNA) in the liver, lung, spleen, and retina of the GFAP-Cre STAT1^f/f^ mice were comparable to those of control mice but were markedly elevated in the brains of the GFAP-Cre STAT1^f/f^ mice (Fig. 3C). Analysis of histological sections confirmed that the susceptibility of GFAP-Cre STAT1^f/f^ mice was associated with the presence of large numbers of replicating tachyzoites. While disease in the GFAP-Cre STAT1^f/f^ mice was associated with the presence of lesions throughout the brain, those that involved the cerebellum were notable (Fig. 3D). These areas were characterized by foci of liquefactive necrosis with cellular and karyorrhectic debris admixed with secondary neutrophilic and histiocytic inflammation. Within these affected regions, there was neuronal and glial necrosis, characterized by hypereosinophilic cytoplasm and karyolysis, and a prominent gliosis. Immunohistochemistry (IHC) analysis using fluorescence or 3,3′-diaminobenzidine (DAB) staining demonstrated the presence of large numbers of infected cells in the CNS compared with control mice (Fig. 3E; also see Fig. S2B in the supplemental material). In addition, staining for GFAP showed that the loss of STAT1 did not compromise the numbers of reactive astrocytes (Fig. 3E). Thus, the susceptibility of the GFAP-Cre STAT1^f/f^ mice is linked with the widespread damage to the CNS caused by extensive areas of parasite replication.

### Loss of STAT in astrocytes promotes cyst formation.

**FIG 4:**
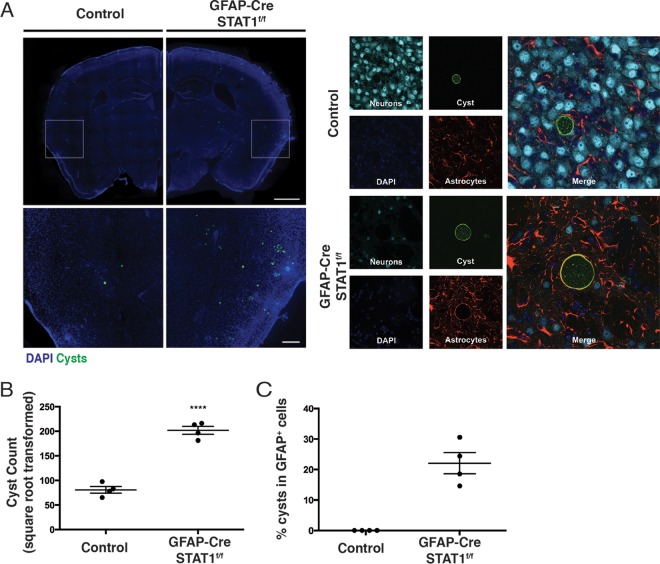
Altered distribution of cysts in GFAP-Cre STAT1^f/f^ mice. (A) (Left) Control and GFAP-Cre STAT1^f/f^ (*n* = 4) mice were infected i.p. with 20 cysts of the ME49 strain, mice were sacrificed at 25 dpi, and brain sections were stained to identify *Toxoplasma* cysts, neurons, and astrocytes, with DAPI being used to visualize host cell nuclei. Forty-micrometer-thick brain sections from 25-dpi control or GFAP-Cre STAT1^f/f^ mice were stained with DAPI (blue) and bradyzoite-specific antigen SAG2X/Y (green). Sections were then examined by fluorescence microscopy. Representative image of whole-brain slices that were reconstructed using a stitched grid of maximum-projection images taken at ×10 magnification. Bar, 1 mm. Enlarged views of boxed regions are shown below. Bar, 200 µm. *n =* 4 mice per genotype, 7 sections per mouse. (Right) Representative images showing a cyst within a neuron in a control brain section (bar, 10 µm) or within a GFAP^+^ astrocyte in GFAP-Cre STAT1^f/f^ mice. (B) The absolute numbers of cyst counts were square root transformed to account for variance between sections and mice (mean ± standard error of the mean; ****, *P* < 0.0001). (C) Percentage (mean ± standard error of the mean) of cysts present in GFAP^+^ host cells in these mice.

Neurons are considered the major host cell that contains the cyst form of *T. gondii* (43); when a specific stain for tissue cysts was performed, there was a significantly increased number of cysts in the brain tissue of GFAP-Cre STAT1^f/f^ mice compared to the low cyst burden observed in control mice (Fig. 4A and B). Moreover, while >99% of the cysts were found in neurons in the control brains, 20 to 30% of cysts in the GFAP-Cre STAT1^f/f^ mice were present in astrocytes, as determined by the pericyst association of GFAP staining with this intracellular form of *T. gondii* (Fig. 4A and C). Together, these results reveal that STAT1 signaling in astrocytes is essential for the local control of parasite replication and suggest that STAT1 mediates astrocyte resistance to cyst formation.

### Impact of astrocytes on local immune responses.

**FIG 5:**
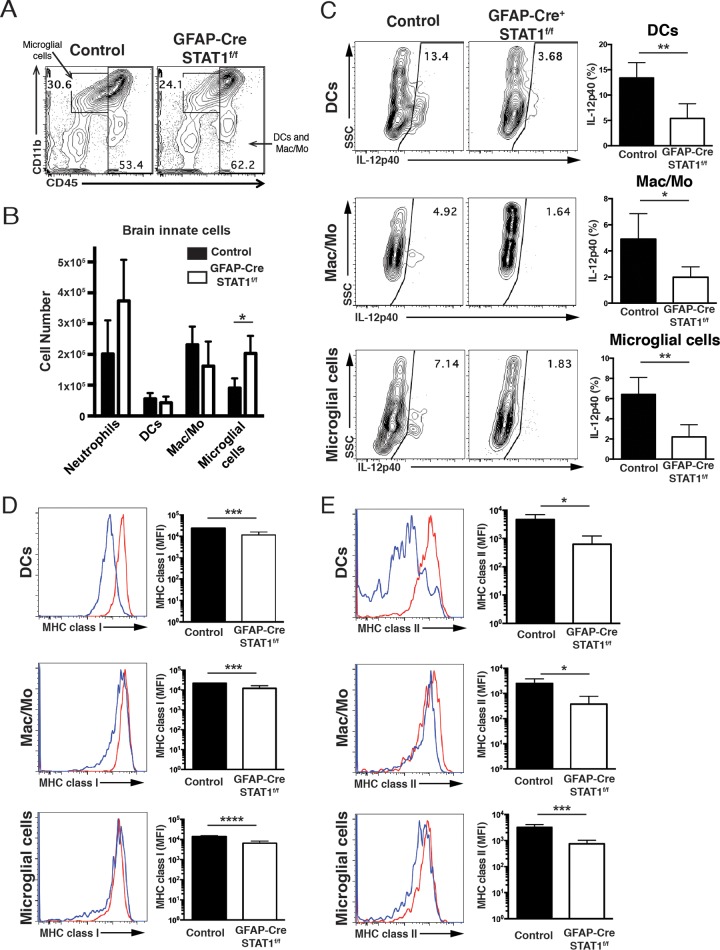
Analysis of the immune populations in infected control and GFAP-Cre STAT1^f/f^ mice. Mice were infected i.p. with 20 cysts of the ME49 strain, and at day 25, the brain was used to prepare mononuclear cell preparations. (A and B) Mononuclear cell preparations from control and GFAP-Cre STAT1^f/f^ mice were prepared, and FACS was used to quantify the numbers of proportions of neutrophils (CD45^hi^, Gr-1^+^, and CD11b^+^), DCs (CD45^hi^, Gr-1^−^, and CD11c^+^), macrophages/monocytes (CD45^hi^, Gr-1^−^, and CD11b^+^), and microglial cells (CD45^int^ and CD11b^+^) gated on dump (CD3, CD19, and NK1.1)^−^ in BMNCs from control and GFAP-Cre STAT1^f/f^ mice. (C) Frequency of IL-12p40 production by microglial cells, DCs, and macrophages/monocytes in the BMNCs. SSC, side scatter. (D and E) Frequency of MHC class I (D) and MHC class II (E) production by DCs, macrophages/monocytes, and microglial cells in the BMNCs. The graphs show means ± standard errors of the means and averages from a total of four mice per group tested at least three times with similar results. *, *P* < 0.05; **, *P* < 0.01; ***, *P* < 0.005.

One possible explanation for the increased susceptibility of the GFAP-Cre STAT1^f/f^ mice is that a defect in the proinflammatory effects of astrocytes could lead to a reduced recruitment of immune populations (T cells and macrophages) that are required to control parasite replication in the CNS (6, 14–17). To assess the effect of STAT1 signaling in astrocytes on the local immune response to *T. gondii*, the absolute number and activation status of T cells, macrophages, and microglia in the brains of GFAP-Cre STAT1^f/f^ mice were compared with those of control mice. First, although astrocytes do not produce inducible nitric oxide synthase (iNOS), macrophages do, and iNOS-deficient mice challenged with *T. gondii* show a similar pattern of susceptibility as the GFAP-Cre STAT1^f/f^ mice (44). However, the overall levels of iNOS expression were comparable between control and GFAP-Cre STAT1^f/f^ brains (see Fig. S3 in the supplemental material), indicating that this antimicrobial pathway was intact. As noted earlier, the numbers of BMNCs in WT and GFAP-Cre STAT1^f/f^ mice at day 10 were comparable, but at 25 dpi in the GFAP-Cre STAT1^f/f^ mice, the absolute number of BMNCs was significantly increased over that of the WT mice (data not shown). Although absolute numbers of infiltrating neutrophils, macrophages/monocytes, and DCs were comparable, the number of microglial cells was significantly increased in the GFAP-Cre STAT1^f/f^ mice (Fig. 5A and B). Unexpectedly, compared with control mice, all of these local populations in infected GFAP-Cre STAT1^f/f^ mice produced reduced levels of IL-12p40 (Fig. 5C) and expressed reduced levels of major histocompatibility complex (MHC) class I and MHC class II but normal levels of the costimulatory molecules CD80 and CD86 (Fig. 5D and E and data not shown).

**FIG 6:**
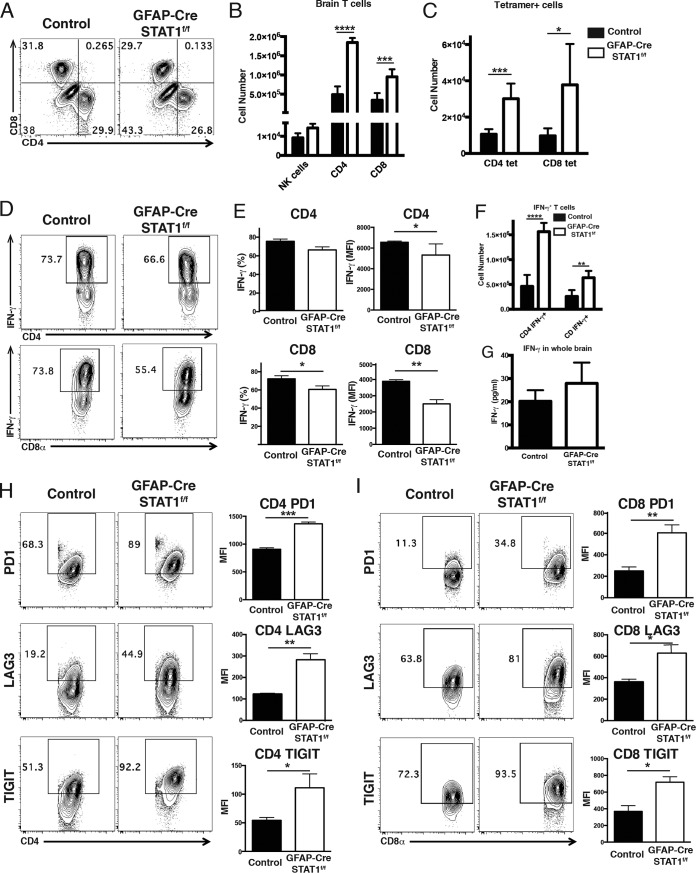
Analysis of production of IFN-γ and expression of inhibitory receptors in T cells during toxoplasmic encephalitis. Control and GFAP-Cre STAT1^f/f^ mice were infected with *T. gondii* and analyzed at 25 dpi. (A and B) Mononuclear cell preparations from control and GFAP-Cre STAT1^f/f^ mice were made, and FACS was used to quantify the numbers of NK cells and CD4^+^ and CD8^+^ T cells. (C) The BMNCs were stained for *T. gondii*-specific T cells using MHC class I tetramer for CD8^+^ T cells and MHC class II tetramer for CD4^+^ T cells, and the absolute number of tetramer^+^ T cells was calculated. (D) The BMNCs were analyzed for the production of IFN-γ^+^ by CD4^+^ and CD8^+^ T cells. (E) Frequency and mean fluorescence intensity (MFI) of IFN-γ production by CD4^+^ and CD8^+^ T cells. (F) Absolute number of IFN-γ^+^ CD4^+^ and CD8^+^ T cells. (G) Whole brain was homogenized in PBS, and supernatant was removed for IFN-γ enzyme-linked immunosorbent assay. (H and I) Comparison of expression levels of PD-1, LAG3, and TIGIT by CD4^+^ (H) and CD8^+^ (I) T cells in the BMNCs. The graphs show means ± standard errors of the means and averages from three to four mice per group, with similar results seen in a repeat experiment. *, *P* < 0.05; **, *P* < 0.01; ***, *P* < 0.005; ****, *P* < 0.001.

Analysis of the lymphocyte responses in the brains of infected mice revealed that the loss of STAT1 resulted in an increased recruitment of T cells but not NK cells (Fig. 6A and B). In both control and GFAP-Cre STAT1^f/f^ mice, the T cells exhibited an activated (Ki-67, granzyme B^+^, T-bet^+^, CD44^hi^ CD62L^low^) phenotype (data not shown), and the use of parasite-specific tetramers for individual class I- and II-restricted epitopes also reflected the increased numbers of parasite-specific T cells present in the GFAP-Cre STAT1^f/f^ mice (Fig. 6C). The use of phorbol myristate acetate (PMA)-ionomycin to stimulate the T cell populations present in the brain showed that the percentage and MFI of IFN-γ^+^ CD4^+^ and CD8^+^ T cells in these mice were reduced (Fig. 6D and E), but the absolute numbers of these IFN-γ^+^ populations were increased in the GFAP-Cre STAT1^f/f^ mice (Fig. 6F). Despite this difference, the levels of total IFN-γ protein detected in soluble brain extract were not different between control and GFAP-Cre STAT1^f/f^ mice (Fig. 6G). Thus, these data indicate that the susceptibility of the GFAP-Cre STAT1^f/f^ mice is not associated with an obvious failure to recruit T cells capable of producing IFN-γ or a global defect in the local production of IFN-γ

The decreased MFI in the production of IFN-γ is reminiscent of chronic CNS infections that include lymphocytic choriomeningitis virus (LCMV) and *T. gondii* where high levels of persistent antigen correlate with repeated activation of T cells, increased expression of inhibitory receptors (PD-1, PD-L1, CTLA4, LAG3, and TIGIT), and a decrease in effector capacity (37, 45–48). Phenotyping of splenic T cells from mice at 25 dpi indicated that the expression of these molecules was largely comparable in control and GFAP-Cre STAT1^f/f^ mice, and in the cervical lymph nodes, T cells expressed similar levels of PD-L1, KLRG1, and CTLA4 (data not shown). However, in the BMNCs from the GFAP-Cre STAT1^f/f^ mice the CD4^+^ and CD8^+^ T cells expressed higher levels of PD-1, LAG3, and TIGIT (Fig. 6H and I). Together, these data indicate that the absence of STAT1 in astrocytes does not impact the initial recruitment of immune cells to the brain, but similarly to other infections, elevated parasite burdens in the CNS are associated with increased T cell recruitment but decreased effector functions.

### Transcriptional profiling of astrocyte responses to IFN-γ and type I interferons.

**FIG 7:**
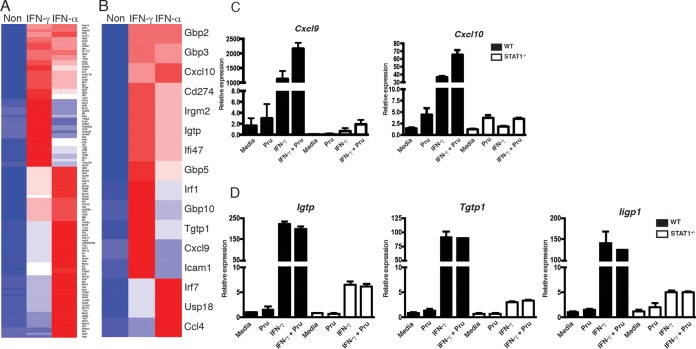
Identification of IFN-γ- and/or IFN-α-inducible genes in primary astrocytes and brain of *T. gondii*-infected GFAP-Cre STAT1^f/f^ mice. Identification of genes that were differentially regulated 2-fold or more compared to unstimulated astrocytes by IFN-γ and IFN-α after 12 h of culture. (A and B) The cluster of genes is shown as a heat map. The heat map color indicates log_2_ expression value. This graph represents average gene expression for replicate arrays. (C and D) Astrocytes were preincubated with IFN-γ for 24 h prior to infection with Pru, and levels of *Cxcl9* and *Cxcl10* (C) and *Igtp*, *Tgtp1*, and *Iigp1* (D) transcripts were normalized to expression of β-actin. The data presented are the mean ± standard error of the mean from three independent experiments.

Stimulation with IFN-γ is known to induce a broad transcriptional program in different cell types (49, 50). However, the IFN-γ-responsive genes in astrocytes are not well characterized, and whether they differ from those induced by type I IFN, which also activate STAT1, is unclear. In an attempt to better understand the impact of IFN-γ on astrocyte function, we utilized a microarray approach to compare the impact of IFN-γ and IFN-α on mouse primary astrocytes. These data sets [accession number GSE67137] revealed that, based on a cutoff of 2-fold change in expression (*P* < 0.05), stimulation of astrocytes with these cytokines led to the upregulation of 122 genes, of which 28 genes were uniquely regulated by IFN-γ but not IFN-α, 48 genes were regulated by IFN-α but not IFN-γ, and 46 genes were similarly regulated by the two IFNs (Fig. 7A). These data sets were used to help identify a subset of genes that were upregulated in response to IFN-α and IFN-γ (*Gbp2*, *Gbp3*, *Cxcl10*, *CD274*, *Irgm2*, *Igtp*, *Ifi47*, and *Gbp5*), IFN-γ alone (*Irf1*, *Gbp10*, *Tgtp1*, *Cxcl9*, and *Icam1*), or IFN-α alone (*Irf7*, *Usp18*, and *Ccl4*) (Fig. 7B) that might be impacted *in vivo* by the loss of STAT1 in astrocytes. This list included multiple p47 GTPases and guanine nucleotide binding proteins (GBPs) associated with control of *T. gondii* (9–11, 51) and also included several chemokines and molecules linked with immunity to this parasite. To validate this approach, WT and STAT1^−/−^ astrocytes were stimulated with *T. gondii*, IFN-γ, or the combination of the two and the levels of transcripts for the canonical STAT1 targets Cxcl9, Cxcl10, and the p47 GTPases (Igtp, Tgtp1, and Iigp1) were assessed. As expected, IFN-γ alone or in combination with *T. gondii* resulted in a marked increase in these targets, but in the absence of STAT1, these were markedly reduced (Fig. 7C and D).

**FIG 8:**
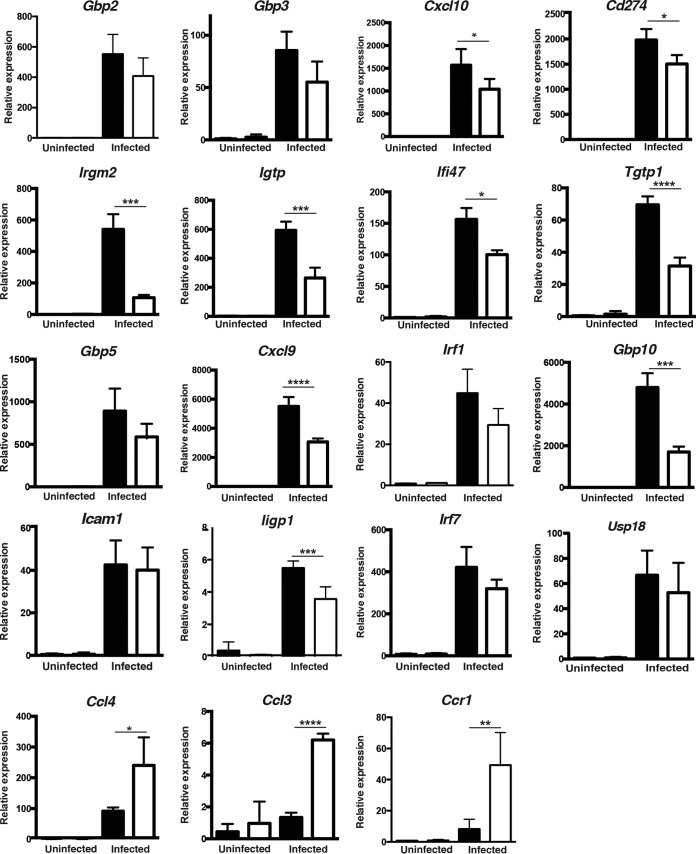
Identification of IFN-γ- and/or IFN-α-inducible genes in primary astrocytes and brain of *T. gondii*-infected GFAP-Cre STAT1^f/f^ mice. RT-PCR was used to estimate the levels of candidate transcripts in the brain of control and GFAP-Cre STAT1^f/f^ mice infected for 25 days. Transcript levels were normalized to the expression of β-actin. These graphs are means ± standard errors of the means of transcript levels from experimental groups of 4 mice each. A paired analysis of the infected control and GFAP-Cre STAT1^f/f^ mice for the interferon-induced targets *Gbp2*, *Gbp3*, *Cxcl10*, *Cd274*, *Irgm2*, *Igtp*, *Ifi47*, *Tgtp1*, *Gbp5*, *Cxcl9*, *Irf1*, and *Gbp10* revealed that this group was significantly different (*P* = 0.045). *, *P* < 0.05; **, *P* < 0.01; ***, *P* < 0.005; ****, *P* < 0.001.

Finally, to provide a global picture of how the loss of STAT1 in astrocytes affected the local response during TE, we performed real-time PCR on the brains of uninfected and infected control and GFAP-Cre STAT1^f/f^ mice to assess the levels of transcripts for the likely IFN-γ and type I IFN targets in astrocytes identified above, as well as the related targets Ligp1, Ccl3, and Ccr1. In this assay, infection in control mice resulted in upregulation of all of these target genes but only a subset were compromised in the GFAP-Cre STAT1^f/f^ mice (Fig. 8). For those targets that were induced by both IFN-α and IFN-γ or IFN-γ alone, the loss of STAT1 in astrocytes was associated with a significant reduction in their levels with one exception, *Icam1*, an adhesion molecule that is widely expressed by many cell types during TE. It was notable that, although we examined only a small group of the genes that were associated uniquely with type I IFNs in astrocytes, the expression of these genes was not reduced in the GFAP-Cre STAT1^f/f^ mice, and indeed for *Ccl3*, the levels of transcripts were actually elevated. The latter result is consistent with the increased levels of the closely related chemokine *Ccl4* and their shared receptor *Ccr1*. Thus, because levels of transcripts for many of the select type I IFN targets were not reduced in the GFAP-Cre STAT1^f/f^ mice, the increased susceptibility of the GFAP-Cre STAT1^f/f^ mice to TE correlates most prominently with reduced expression of p47 GTPases and GBPs (molecules that contribute directly to astrocyte control of *T. gondii*) and with reduced production of the IFN-γ-inducible chemokines produced by *Cxcl9* and *Cxcl10*.

## DISCUSSION

Activation and proliferation of astrocytes represent a common feature of many insults, including infection, that lead to tissue damage in the brain. The generation of transgenic mice in which the GFAP promoter was used to drive overexpression of cytokines or alter signaling pathways has highlighted the role of astrocytes in limiting immune infiltration to promote healing (52–54). In current models, this extensive gliosis during TE provides a physical barrier to limit tissue damage and isolate areas of pathogen replication (18, 20, 21), but whether astrocytes contribute directly to parasite control *in vivo* has been difficult to address experimentally. Here, the finding that the GFAP-Cre STAT1^f/f^ mice fail to control parasite replication in the CNS provides the first *in vivo* evidence that astrocytes have direct antimicrobial activity that mediates local control of *T. gondii*. This genetic approach has been used extensively to address astrocyte function but can also cause gene deletion in a subset of microglial cells, neurons, and neural stem cells (33, 55, 56), although we found no evidence of STAT1 deletion in these other cell types or that it compromised antimicrobial activities in these populations. For example, the overall levels of iNOS expression were comparable between control and GFAP-Cre STAT1^f/f^ brains. Furthermore, since IFN-γ does not activate neurons to inhibit parasite replication (57), the increased parasite burden of the GFAP-Cre STAT1^f/f^ mice is consistent with a key role for IFN-γ in promoting cell intrinsic pathways in astrocytes to control parasite replication (58–60).

*In vitro* studies in astrocytes have established that several of the p47 GTPases can be recruited to the parasitophorous vacuole, and the absence of IIGP1 or IGTP results in partial loss of the ability of IFN-γ to restrict growth of *T. gondii* in these cells (10, 61). A second group of proteins, the p65 GTPases, are part of this machinery, and two recent studies highlighted their role in resistance to *T. gondii* (12, 13). Thus, the reduced expression of transcripts for these families observed in the GFAP-Cre STAT1^f/f^ mice supports the idea that their susceptibility is a consequence of the inability of IFN-γ to activate astrocytes to limit the growth of *T. gondii*. These data have to be interpreted with care as multiple cytokines can activate STAT1, and our data confirmed the ability of type I interferons to activate STAT1 and illustrated that IFN-γ and the type I IFNs have unique as well as overlapping effects on the transcriptional profile of astrocytes. However, our preliminary studies suggest that, whereas the neutralization of IFN-γ in chronically infected mice results in increased parasite replication, this is not the case for blockade of the type I IFN receptor (S. Hidano and C. A. Hunter, unpublished observations). The latter finding is consistent with the observation that the levels of transcripts for the type I IFN targets *Irf7*, *Usp18*, and *Ccl4* are not decreased in the infected GFAP-Cre STAT1^f/f^ mice.

One unanticipated observation was that the loss of STAT1 in astrocytes resulted in altered cellular distribution of the cyst stage. In infected mice, under normal circumstances, this stage is found predominantly in neurons (43), whereas in GFAP-Cre STAT1^f/f^ mice this latent form was increased in numbers and readily detected in astrocytes. Similarly, in chronically infected WT mice, treatment with anti-IFN-γ resulted in increased levels of parasite replication accompanied by elevated cyst numbers (8, 22). Although the distribution of cysts in different cell types was not assessed in the latter studies, the presence of cysts in astrocytes in the GFAP-Cre STAT1^f/f^ mice is unlikely to be a consequence of the higher parasite burden. Thus, infected GFAP-Cre gp130^f/f^ mice have increased parasite burden in the CNS but no significant increase in cyst numbers (20). Together, these observations suggest that the activation of STAT1 in astrocytes inhibits cyst formation. Alternatively, there are host cell pathways, such as signaling through CD73, which promote cyst formation (62), and STAT1 signaling in astrocytes may disrupt these events. This idea stands in contrast to previous *in vitro* studies in which IFN-γ was used to promote cyst formation in astrocytes (63), and additional approaches are needed to directly address the events that limit cyst formation in astrocytes.

Given the role for astrocytes in the control of inflammation, it seemed likely that the GFAP-Cre STAT1^f/f^ mice would display additional phenotypes that were related to their regulatory properties. For example, the absence of the cytokine receptor gp130 on astrocytes results in a widespread loss of these cells during TE (20), and because gp130 signaling activates STAT1, it was possible that the GFAP-Cre STAT1^f/f^ mice would have a similar phenotype. However, we found that IL-6 (one of the major inflammatory cytokines that utilizes gp130) did not activate STAT1 in astrocytes, and the histological analysis and staining patterns for GFAP in the GFAP-Cre STAT1^f/f^ mice revealed extensive gliosis and astrocytic responses to areas of parasite replication. These results imply that other signaling pathways employed by gp130, such as STAT3 and mitogen-activated protein kinase (MAPK), mediate the neuroprotective effects of astrocytes. Astrocytes are also a prominent chemokine source linked to recruitment of immune cells during TE (6), and the reduced levels of *Ccxl9* and *Ccxl10* in the CNS of the GFAP-Cre STAT1^f/f^ mice are consistent with that idea. However, the recruitment of inflammatory macrophages, DC populations, and T cells in the GFAP-Cre STAT1^f/f^ mice did not appear compromised. This may be a consequence of the elevated levels of CCL3, a chemokine that promotes CD8^+^ T cell effector function and migration in the CNS (64). Indeed, *T. gondii* can stimulate neurons to produce CCL3 (57), and the increased parasite burden in the GFAP-Cre STAT1^f/f^ mice may lead to an increased reliance on this pathway to recruit inflammatory populations. Nevertheless, despite normal initial T cell responses in the CNS, at later time points the T cells in the brains of the GFAP-Cre STAT1^f/f^ mice had a small but reproducible reduction in their ability to produce IFN-γ and increased expression of inhibitory receptors. Since the disease in the GFAP-Cre STAT1^f/f^ mice resembles that in chronically infected mice with severe immune deficiencies (i.e., treated with neutralizing antibodies specific for IFN-γ or depleted of both CD4^+^ and CD8^+^ T cells), it seems unlikely that the relatively modest changes in effector function would be sufficient to explain the high parasite burden in the GFAP-Cre STAT1^f/f^ mice. Rather, we favor a scenario in which the absence of STAT1 in astrocytes leads to their failure to control parasite replication and this increased antigen load would lead to persistently stimulated parasite-specific T cells that display an “exhausted” phenotype. This is supported by reports that correlate increased T cell expression of PD-1 with the progression of TE or which showed that blockade of PD-1/PD-L1 resulted in increased effector responses and better control of *T. gondii* (37, 48).

Over the last 30 years, there has been an improved understanding of how the immune system operates within the CNS to control *T. gondii*. In particular, local perforin-mediated cytotoxic T lymphocyte (CTL) activity and production of IFN-γ and tumor necrosis factor alpha (TNF-α), which promote macrophage antimicrobial effector mechanisms, have key roles in the control of *T. gondii* (44, 65–68). Similarly, stimulation through CD40 can promote the control of *T. gondii* (69, 70), and in astrocytes, this pathway is STAT1 independent (Hidano and Hunter, unpublished). Nevertheless, in the GFAP-Cre STAT1^f/f^ mice, these other antiparasite effector mechanisms appear largely intact but are not sufficient for long-term parasite control. This diverse literature highlights the need for an integrated immune response that engages multiple effector pathways for the control of *T. gondii* in the CNS. There has been a longstanding interest in using this information to design strategies that would enhance the ability of the immune system to control *T. gondii* in patients with underlying T cell defects. Knowing that STAT1 is important in astrocyte immune functions under normal circumstances should provide novel leads for therapeutic strategies to eradicate the cyst stage and better manage TE without compromising the neuroprotective effects of these glia.

## MATERIALS AND METHODS

### Culture of primary astrocytes.

Astrocytes were isolated from mixed glial cultures derived from the cerebral cortex of 1- to 3-day-old neonatal mice, as previously described (9, 71). After 12 days of culture, astrocytes (>90% GFAP^+^) were plated at a density of 1 × 10^5^ cells/cm^2^ in 12-well plates. To assess the ability of IFN-γ to limit parasite replication, astrocytes were stimulated with 100 U/ml IFN-γ for 24 h prior to infection. Cultures were washed, and tachyzoites of the Prugniaud (Pru) strain, Pru-green fluorescent protein (GFP) strain, or Pru-Venusluc strain of *T. gondii* were added at a ratio of 5 Pru cells to 1 host cell for 20 h. The Pru-Venusluc strain contains a fusion protein of Venus (a modified version of YFP) and *Photinus pyralis* luciferase and was obtained from M. Yamamoto (72). The percentage of infected cells was assessed microscopically by Hema3 (Fisher Scientific, Kalamazoo, MI) staining. To assess STAT phosphorylation, astrocytes were incubated with IFN-γ and infected with Pru at a ratio of 1 Pru cell to 1 host cell for 1 h. For universal type I IFN (PBL Interferon Source, Piscataway, NJ), IL-6 (BioLegend, San Diego, CA), and IL-27 (Amgen, Thousand Oaks, CA), cells were stimulated for 1 h. Astrocytes were immediately fixed on ice in 4% paraformaldehyde (PFA) for 20 min and were permeabilized in 90% methanol on ice. Staining was performed in Fc block with anti-pSTAT1 (pY701) (BD Bioscience, Franklin Lakes, NJ).

### Mice and infections.

STAT1^−/−^ mice and GFAP-Cre STAT1^f/f^ mice were bred in the University Laboratory Animal Resources facilities at the University of Pennsylvania. hGFAP-Cre transgenic mice [Tg(GFAP-cre)25Mes] (33) and STAT1^−/−^ mice (73) were obtained from the Jackson Laboratory (Bar Harbor, ME), and STAT1^f/f^ mice were generated as previously described (74). Age- and sex-matched conditional knockout mice were used in experiments, with STAT1^f/f^ mouse littermates serving as controls. For infections, cysts of the ME49 strain were isolated from chronically infected CBA mice and mice were infected with 20 cysts intraperitoneally (i.p.). All procedures were performed in accordance with the guidelines of the University of Pennsylvania Institutional Animal Care and Use Committee.

### Real-time PCR.

Real-time PCR was utilized to quantify parasite DNA as previously described (75). Briefly, DNA was purified from approximately 50 mg of tissue using the High Pure PCR template preparation kit (Roche, Mannheim, Germany). Primers for the *T. gondii* B1 repeat region were used to quantify the amount of parasite DNA from 500 ng of DNA. Total RNA was isolated with the Trizol reagent, and first-strand cDNAs were synthesized using oligo(dT) primers and the Superscript reverse transcription-PCR (RT-PCR) kit (Invitrogen, Carlsbad, CA). Primers used were QuantiTect primers (Qiagen, Valencia, CA), and primer sequences are listed in the supplemental material. cDNAs were amplified using Power SYBR Green PCR master mix and a 7500 Fast real-time PCR system. Analysis was performed with system software, v1.3.1 (Applied Biosystems, Warrington, United Kingdom).

### Analysis of immune responses to *T. gondii.*

Splenocytes were dissociated and subjected to hypotonic red blood cell lysis to generate a single-cell suspension. BMNCs were extracted from the CNS following treatment of this tissue with collagenase-dispase and DNase I and then isolated by density gradient centrifugation using Percoll as previously described (75). Single-cell suspensions were stained in fluorescence-activated cell sorting (FACS) buffer (0.5% bovine serum albumin [BSA], 2 mM EDTA in phosphate-buffered saline [PBS]) with Fc block containing LIVE/DEAD Fixable Aqua dead cell marker (Invitrogen), with a combination of cell surface antibody markers. Cell surface staining was used with a combination of fluorescein isothiocyanate (FITC), phycoerythrin (PE), PE-CF594, peridinin chlorophyll protein (PerCP)-Cy5.5, PE-Cy7, Alexa Fluor 700, allophycocyanin (APC)-Alexa Fluor 780, APC, and APC-Cy7. The conjugated and unconjugated antibodies specific to the following antigens (CD3e, 2C11; CD19, eBio1D3; NK1.1, PK136; CD45, 30-F11; CD11b, M1/70; CD11c, N418; MHC class II, M5/114.15.2; CD4, RM4-5; CD8a, GK1.5; DX5, DX5; Ly6G, RBC-8C5; Ly6C, HK1.4; PD1, J43; LAG3, eBioC9B7W; TIGIT, MBSA43) were purchased from BD Biosciences (San Jose, CA), BioLegend (San Diego, CA), and eBioscience (San Diego, CA). For cytokine production, cells were plated at a cell density of 1 × 10^6^ cells per well in 96-well plates and were assayed using cells stimulated for 4 h with or without PMA and ionomycin in the presence of brefeldin A and monensin. These cells were stained for surface markers and fixed with 4% PFA in PBS. Intracellular IFN-γ (XMG1.2) and IL-12p40 (C15.6) were detected by staining in 0.5% saponin buffer (Sigma, St. Louis, MO). Data were collected on a BD LSRFortessa cell analyzer (BD Bioscience) and analyzed using FlowJo software (Tree Star, Ashland, OR).

### Histology and IHC.

To detect the presence of *T. gondii* in paraffin sections, tissues were fixed overnight in 10% formalin (Sigma) and embedded in paraffin, and 10-µm sections were prepared for immunohistochemistry (IHC) as previously described. For frozen sections, brains were bisected and frozen with optimal cutting temperature (OCT) solution (Tissue-Tek, Torrance, CA), serial sagittal sections were prepared, and frozen sections were fixed in 100% acetone. Primary astrocytes were plated at a density of 1 × 10^5^ cells/cm^2^ in 12-well plates, fixed with 90% methanol, and then blocked in 2% normal goat serum (NGS) prior to incubation with antibodies for GFAP (2A5; Dako, Carpinteria, CA; 1:400), STAT1 (E-23; Santa Cruz Biotechnology, Dallas, TX; 1:200), iNOS (ab15323; Abcam, Cambridge, MA; 1:200), CD11b (M1/70; BD Bioscience; 1:200), and IGTP (BD Bioscience; 1:200), followed with appropriate secondary antibodies conjugated to Alexa Fluor 488 or Alexa Fluor 594. DAPI (4′,6-diamidino-2-phenylindole) (Invitrogen) was used to visualize nuclei. Samples were mounted in ProLong Gold. Images were collected on a Nikon E600 fluorescence microscope and analyzed using NIS-Element (Nikon, Tokyo, Japan).

For whole mounts, brains were collected and fixed for 24 h in PFA and then immersed in 30% sucrose in PBS before the preparation of 40-µm-thick coronal brain sections. To detect *Toxoplasma* cysts, brain sections were incubated with fluorescein-labeled *Dolichos biflorus* agglutinin (Vector Laboratories; FL-1031; 1:500), a lectin that binds sugar moieties in the cyst wall (76), and immunostained with antibodies specific for GFAP (Dako; Z0334; 1:200) and neurons using a cocktail of biotinylated anti-NeuN (Millipore; MAB3778; 1:200), mouse anti-MAP2 (Sigma; M2320; 1:2,000), and chicken antineurofilament (Abcam; ab4680; 1:20,000). Where appropriate, species-appropriate Alexa Fluor-conjugated or Cy5-streptavidin secondary antibodies were used (Invitrogen; A31556, A21236, A21449, and 434316), and sections were mounted with Vectashield Hardset mounting medium (Vector; H-1400). Images were obtained using a 63× oil lens on a Leica SP5-II confocal microscope or a 10× lens on an upright fluorescence microscope (Deltavision RT deconvolution fluorescence). Images were analyzed using ImageJ software or Adobe Photoshop CS3.

### Microarray analysis.

Whole-genome expression microarray analysis was performed as previously described (77). Microarrays and data analyses were carried out as previously described (78). Briefly, total RNA was isolated from untreated WT primary astrocytes or cells treated with 10 ng/ml of either recombinant IFN-γ or universal type I interferon (PBL Assay Science) for 12 h, and then biotin-labeled cRNA was generated. Illumina MouseRef-8 version 2 expression BeadChips were hybridized with cRNA and scanned, and images were converted to raw expression values. Data analyses were carried out using the statistical computing environment R (v3.0.2), the Bioconductor suite of packages for R, and RStudio (v0.97). Probe sets that were differentially regulated ≥2-fold (*P* ≤ 0.05) after controlling for multiple testing using the Bonferroni-Hochberg method were used for heat map generation. Genes were defined as being selectively induced if they were expressed ≥2-fold by IFN-γ treatment relative to control but not by type I interferon treatment or vice versa. Genes were defined as inducible by both cytokines if their expression was increased >2-fold by both treatments.

### Statistics.

Statistical significance was determined using a two-tailed unpaired Student *t* test, which was performed using Prism 6 software (GraphPad Software, La Jolla, CA). Significance of survival curves was assessed using Kaplan-Meier survival curves. Where appropriate, a paired analysis was used to assess changes in levels of IFN-γ-induced genes. Error bars indicate the standard deviations of the means: *, *P* < 0.05; **, *P* < 0.01; ***, *P* < 0.001; ****, *P* < 0.0001.

### Accession number(s).

Nonnormalized, non-background-subtracted raw data have been deposited in the Gene Expression Omnibus (GEO) database for public access (accession number GSE67137).

## SUPPLEMENTAL MATERIAL

Figure S1  Reduced STAT1 expression in primary astrocytes from GFAP-Cre STAT^f/f^ mice. (A and B) Primary astrocytes from control or GFAP-Cre STAT1^f/f^ mice were stained for anti-STAT1 (green) and anti-GFAP (red) antibodies, with DAPI (blue) as a nuclear counterstain, in unstimulated cells (A) or cells stimulated with 100 U/ml IFN-γ for 1 h (B). Bar, 20 µm. (C) Microglial cells isolated from the brain of adult control or GFAP-Cre STAT1^f/f^ mice were stimulated with 100 U/ml IFN-γ for 1 h and then assayed for STAT1 phosphorylation by FACS. (D) IFN-γ-mediated growth inhibition and intracellular killing of *T. gondii* in the control and GFAP-Cre STAT1^f/f^ primary astrocytes. Error bars indicate the standard deviations of the means; **, *P* < 0.01. (E) The brain section from control or GFAP-Cre STAT1^f/f^ mice at 25 dpi was stained for anti-STAT1 (green) and anti-GFAP (red) antibodies, with DAPI (blue) as a nuclear counterstain. Bar, 20 µm. Download Figure S1, TIF file, 0.2 MB

Figure S2  Levels of *T. gondii* in multiple tissues from control and GFAP-Cre STAT1^f/f^ mice. The liver, lung, spleen, brain, and retina were stained for anti-ME49 antibody at 10 dpi (A) or 25 dpi (B). Representative sections are shown. Bar, 100 µm. Download Figure S2, TIF file, 0.2 MB

Figure S3  iNOS production in brain of control and GFAP-Cre STAT1^f/f^ mice. The brain section from control or GFAP-Cre STAT1^f/f^ mice at 25 dpi was stained for anti-CD11b (green) and iNOS (red) antibodies, with DAPI (blue) as a nuclear counterstain. Bar, 20 µm. Download Figure S3, TIF file, 0.1 MB
