# Supplementary figures and images for: STAT1 Signaling in Astrocytes Is Essential for Control of Infection in the Central Nervous System
Source: mBio. 2016 Nov 8;7(6):e01881-16. doi: 10.1128/mBio.01881-16 (PMC5101356; doi:10.1128/mBio.01881-16)

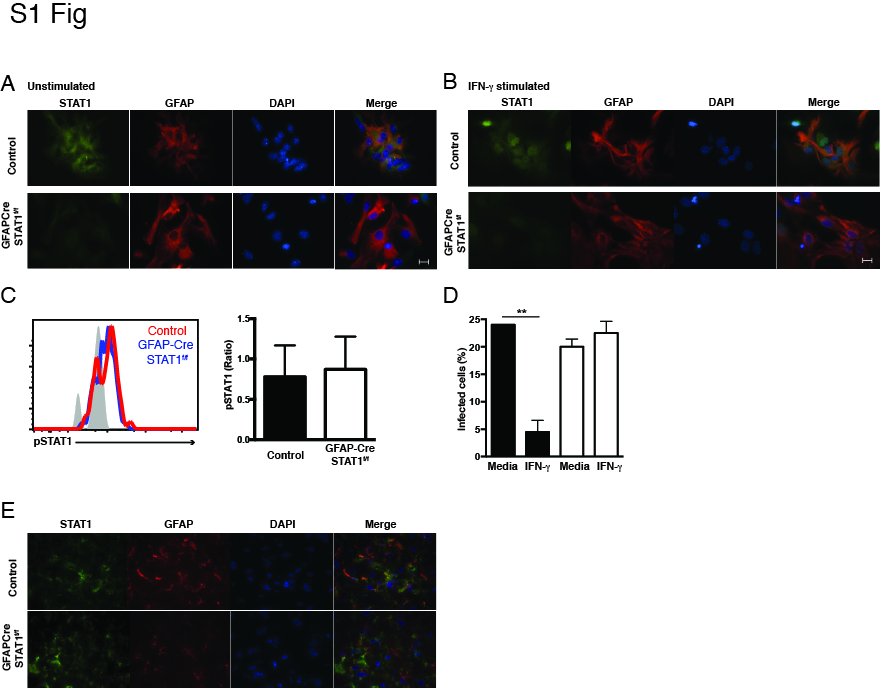

Supplement: Figure S1 — Reduced STAT1 expression in primary astrocytes from GFAP-Cre STATf/f mice. (A and B) Primary astrocytes from control or GFAP-Cre STAT1f/f mice were stained for anti-STAT1 (green) and anti-GFAP (red) antibodies, with DAPI (blue) as a nuclear counterstain, in unstimulated cells (A) or cells stimulated with 100 U/ml IFN-γ for 1 h (B). Bar, 20 µm. (C) Microglial cells isolated from the brain of adult control or GFAP-Cre STAT1f/f mice were stimulated with 100 U/ml IFN-γ for 1 h and then assayed for STAT1 phosphorylation by FACS. (D) IFN-γ-mediated growth inhibition and intracellular killing of T. gondii in the control and GFAP-Cre STAT1f/f primary astrocytes. Error bars indicate the standard deviations of the means; **, P < 0.01. (E) The brain section from control or GFAP-Cre STAT1f/f mice at 25 dpi was stained for anti-STAT1 (green) and anti-GFAP (red) antibodies, with DAPI (blue) as a nuclear counterstain. Bar, 20 µm. Download [file mbo006163060sf1.tif]

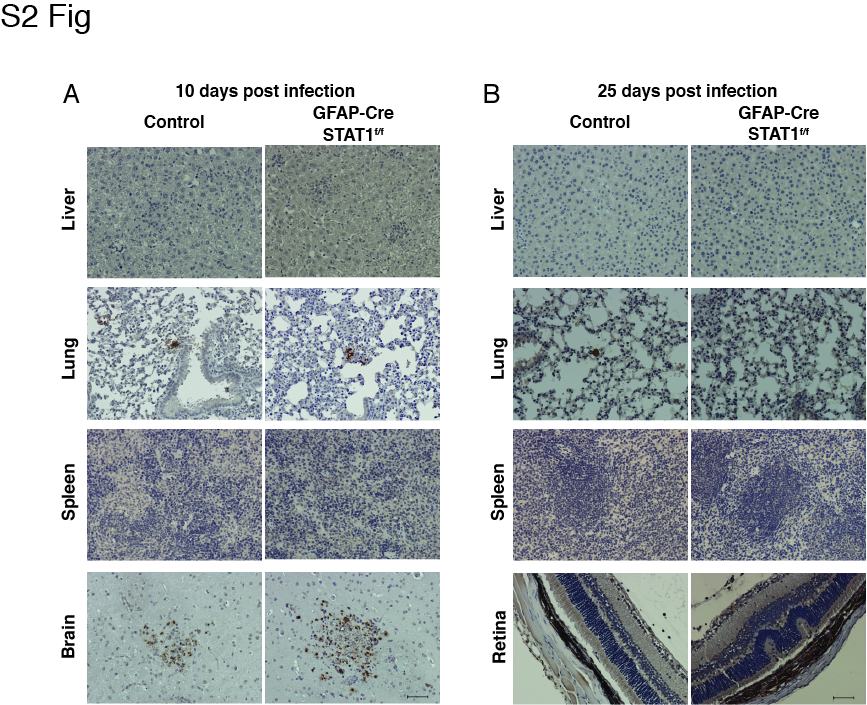

Supplement: Figure S2 — Levels of T. gondii in multiple tissues from control and GFAP-Cre STAT1f/f mice. The liver, lung, spleen, brain, and retina were stained for anti-ME49 antibody at 10 dpi (A) or 25 dpi (B). Representative sections are shown. Bar, 100 µm. Download [file mbo006163060sf2.tif]

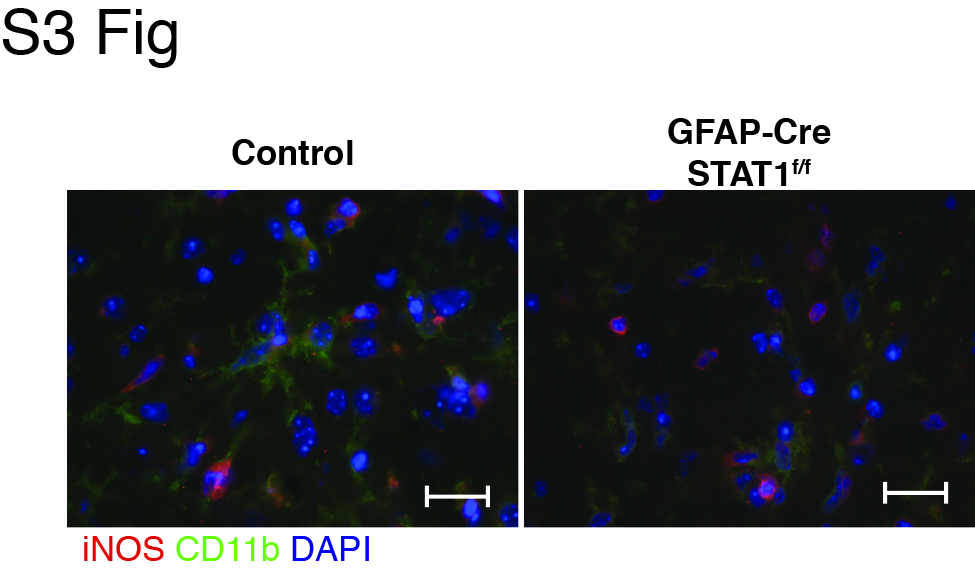

Supplement: Figure S3 — iNOS production in brain of control and GFAP-Cre STAT1f/f mice. The brain section from control or GFAP-Cre STAT1f/f mice at 25 dpi was stained for anti-CD11b (green) and iNOS (red) antibodies, with DAPI (blue) as a nuclear counterstain. Bar, 20 µm. Download [file mbo006163060sf3.tif]
